# Versican—A Critical Extracellular Matrix Regulator of Immunity and Inflammation

**DOI:** 10.3389/fimmu.2020.00512

**Published:** 2020-03-24

**Authors:** Thomas N. Wight, Inkyung Kang, Stephen P. Evanko, Ingrid A. Harten, Mary Y. Chang, Oliver M. T. Pearce, Carys E. Allen, Charles W. Frevert

**Affiliations:** ^1^Matrix Biology Program, Benaroya Research Institute at Virginia Mason, Seattle, WA, United States; ^2^Division of Pulmonary/Critical Care Medicine, Center for Lung Biology, University of Washington School of Medicine, Seattle, WA, United States; ^3^Centre for the Tumour Microenvironment, Barts Cancer Institute, Queen Mary University of London, London, United Kingdom

**Keywords:** versican, hyaluronan, immunity, inflammation, macrophages, T lymphocytes

## Abstract

The extracellular matrix (ECM) proteoglycan, versican increases along with other ECM versican binding molecules such as hyaluronan, tumor necrosis factor stimulated gene-6 (TSG-6), and inter alpha trypsin inhibitor (IαI) during inflammation in a number of different diseases such as cardiovascular and lung disease, autoimmune diseases, and several different cancers. These interactions form stable scaffolds which can act as “landing strips” for inflammatory cells as they invade tissue from the circulation. The increase in versican is often coincident with the invasion of leukocytes early in the inflammatory process. Versican interacts with inflammatory cells either indirectly via hyaluronan or directly via receptors such as CD44, P-selectin glycoprotein ligand-1 (PSGL-1), and toll-like receptors (TLRs) present on the surface of immune and non-immune cells. These interactions activate signaling pathways that promote the synthesis and secretion of inflammatory cytokines such as TNFα, IL-6, and NFκB. Versican also influences inflammation by interacting with a variety of growth factors and cytokines involved in regulating inflammation thereby influencing their bioavailability and bioactivity. Versican is produced by multiple cell types involved in the inflammatory process. Conditional total knockout of versican in a mouse model of lung inflammation demonstrated significant reduction in leukocyte invasion into the lung and reduced inflammatory cytokine expression. While versican produced by stromal cells tends to be pro-inflammatory, versican expressed by myeloid cells can create anti-inflammatory and immunosuppressive microenvironments. Inflammation in the tumor microenvironment often contains elevated levels of versican. Perturbing the accumulation of versican in tumors can inhibit inflammation and tumor progression in some cancers. Thus versican, as a component of the ECM impacts immunity and inflammation through regulating immune cell trafficking and activation. Versican is emerging as a potential target in the control of inflammation in a number of different diseases.

## Introduction

Inflammation occurs during tissue infection or injury and involves the migration of leukocytes out of the blood vessels and into damaged areas of tissue (1, 2). Inflammation is driven by receptors on the surface of immune and non-immune cells (pattern recognition receptors, PRR) (1). PRRs recognize three classes of molecular patterns resulting from either pathogens generated by infectious agents such as virus and bacteria (pathogen-associated molecular patterns or PAMPs), molecules released from damaged tissues (damage-associated molecular patterns or DAMPs), or from molecular patterns on “self” tissues, often upregulated during malignancy (self-associated molecular patterns, SAMPs) (3). Activation of these receptors initiates an inflammatory response involving inflammatory cytokine production and recruitment of leukocytes. While inflammation is important in repairing tissue after insult, it often results in an exacerbation of tissue injury and promotion of disease.

Recent studies have indicated an important role for the ECM in the inflammatory response (4–10). Leukocytes cross the endothelial barrier and interact with the ECM which influences their adhesion, retention, migration, and activation. Leukocyte trafficking and localization are critical to events associated with the immune response. Specific components of the ECM can act as DAMPs or matrikines (11) promoting inflammatory cytokine synthesis and release by immune and non-immune cells (4, 12, 13). Proteoglycans, as components of the ECM, play a key part in providing intrinsic signals needed to coordinate critical events in the inflammatory cascade (4–6, 10, 14–23).

We have been interested in versican, which is a chondroitin sulfate proteoglycan (CSPG) and a member of the hyalectin family of ECM components (24), as one of the principal drivers of immunity and inflammation in a variety of different diseases, such as cardiovascular and lung disease, autoimmune diseases, and several different cancers. Interestingly, like many other ECM components, versican has “two faces”—functioning both in a pro- and an anti-inflammatory manner. The goal of this review will be to highlight the involvement of versican as a component in inflammation, discuss its role in recruiting and activating leukocytes, and provide examples and possible mechanisms by which this “versatile” ECM molecule can exhibit both pro- and anti-inflammatory properties.

## Versican

The expression and accumulation of versican, a large ECM proteoglycan, increases dramatically during inflammation in most diseases [reviewed in (4–6, 10, 23, 25, 26)]. Versican, named for its versatility in being a highly interactive molecule (27), is encoded from a single gene locus on chromosome 5q14.3 in humans (28) and its full-length isoform shares 76% nucleotide and 62% amino acid sequence identity between mouse and human.

There are at least five different isoforms of versican, V0, V1, V2, V3, V4, due to the alternative splicing of the major exons that code for the attachment regions for the chondroitin sulfate (CS) glycosaminoglycans (GAG) in the core protein (27, 29–33). Four of these isoforms contain CS GAGs that are attached by covalent linkage to the core protein, while one of the isoforms, V3, contains no GAGs due to the splicing together of the N- and C- terminal regions. V0, V1, V2, and V3 differ in the size of the core proteins and the size and number of the GAG chains. Additional variation within these isoforms has also been observed with an alternatively spliced C-terminus “Vint tail” (33).

The calculated molecular masses from cDNA sequencing studies for human versican core proteins are 370 kDa for V0, 262 kDa for V1, and 72 kDa for V3. These theoretical values are significantly lower than deduced from SDS PAGE gels where V0 migrates at around 550 kDa, and V1 around 500 kDa after chondroitin ABC lyase digestion (34). These differences are due to the high content of O- and N-linked oligosaccharides associated with the versican core protein (34–36). Interestingly, the different isoforms exhibit functional differences regarding their impact on cell phenotype, such as the ability of V1 to promote proliferation and inhibit apoptosis, while V2 exhibits antiproliferative activity (37, 38). In contrast, V3 regulates ECM assembly and inhibits cell proliferation and migration (39–45). A new V5 isoform has been recently described and shown to be expressed by injured rat neurons (46). While the biological importance of the “Vint tail” has yet to be elucidated, it likely provides fine tuning of interactions with the G3 region of the molecule. Thus, the different molecular domains of versican make the biology of this molecule quite complex given that both the alternative splicing of the mRNA as well as the controlled degradation of the intact versican molecule generate many different molecular forms which themselves can have their own effects on key events associated with immunity and inflammation. Much work remains to be done to sort out these complex interactions and their effects on the biology of this highly interactive molecule.

There is variation in the number, size, and composition of the CS chains attached to the different isoforms of versican. For example, consensus sequence for the number of CS attachment sites on the core protein of human versican reveals 17–23 sites for V0, 12–15 for V1, 5–8 for V2, and 0 for V3 (36, 47). Growth factors that increase during inflammation such as platelet-derived growth factor (PDGF) and transforming growth factor-beta (TGFβ) increase CS chains length and alter CS composition, impacting the ability of versican to interact with other molecules (35, 48–50). The CS isolated from versican interacts with inflammatory cytokines and impacts cytokine activity (51) [also see reviews (52, 53)]. Interestingly, the machinery and signaling pathways that control the composition and elongation of the CS chain differ from those controlling the transcription of versican core protein synthesis (48). For example, we found that stimulation of versican core protein synthesis by PDGF in non-human primate arterial smooth muscle cells encoded both the PKC and ERK pathways, whereas the elongation of the CS chains attached to the versican core protein was PKC dependent, but ERK independent. Since GAG chain length is a critical factor in determining the interactive nature of versican as well as other proteoglycans (54), targeting the pathways that regulate GAG chain elongation (55) may be a useful therapeutic approach to alter the immune and inflammatory properties of this ECM component.

Versican is synthesized by many different types of cells, including epithelial, endothelial, and stromal cells as well as leukocytes. This synthesis is regulated by a host of proinflammatory cytokines and growth factors [reviewed in (26, 56)]. Versican expression is regulated through two signaling pathways. Rhamani et al. first described the role of the canonical Wnt/β-catenin/T-cell factor (TCF) pathway in regulating versican expression in airway smooth muscle cells (57–59). Using polyinosinic-polycytidylic acid (poly I:C) and lipopolysaccharide (LPS) to activate TLR3 and TLR4, respectively, we showed that the signaling cascade that includes TLR3 or TLR4, the TLR adaptor molecule, Trif, type I interferons (IFNs), and the type I IFN receptor (IFNAR1), increases versican expression by mouse macrophages, which implicates versican as an IFN-stimulated gene (60). Versican synthesis is also controlled by several different miRNAs which are modulated during inflammation (61–63).

The degradation of versican is affected by several different families of proteases that increase during inflammation. Such proteases include matrix metalloproteinases (MMPs), i.e., MMP-1,−2,−3,−7, and−9 (64–66), serine protease plasmin (67), and at least five ADAMTS (a disintegrin and metalloproteinase with thrombospondin motifs) MMPs, specifically ADAMTS-1,−4,−5,−9, and−20 [see reviews (12, 13, 68–70)]. Cleavage of versican by ADAMTS-1,−4,−5, and−9 leads to production of an amino-terminal fragment, termed versikine, that can be detected using an antibody recognizing the neoepitope sequence DPEAAE (DPE) (12, 71, 72). Fragments such as versikine can act as DAMPs, interacting with immune and non-immune cells stimulating pro- and anti-inflammatory cytokine release and a modified immune response in both human and murine models (13, 73–75). Thus, many mechanisms are in place to regulate the expression and degradation of versican during the inflammatory process which ultimately influence key cellular events including cell adhesion, proliferation, migration, and ECM remodeling.

## Versican: A Component of the Inflammatory Response

**Figure 1:**
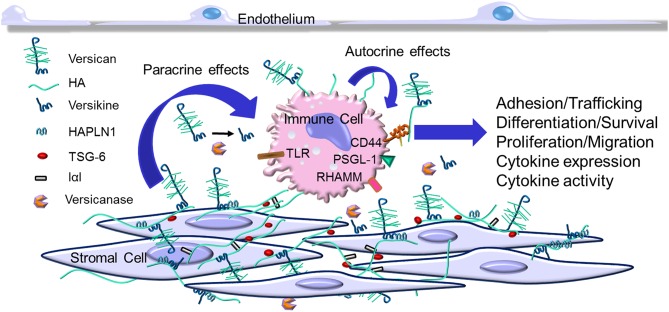
Versican increases in the extracellular matrix (ECM) as part of the early inflammatory response. Immune cells invade the tissue from the blood stream and interact with specific ECM components including versican, versican fragments such as versikine (generated by versicanases), and other ECM components that associate with versican. This interaction may involve receptors on the immune cells such as CD44, P-selectin glycoprotein 1 (PSGL-1), receptor for hyaluronan-mediated motility (RHAMM), and toll-like receptors (TLRs). This interaction impacts several aspects of immune cell activation as part of the immune and inflammatory response.

Versican is essential during development (76, 77) and it is now becoming apparent that it is an important component of the tissue inflammation caused by infection and tissue injury (10). Versican accumulates as part of the early inflammatory response in a number of human diseases often associated with the invasion of leukocytes including those in the vascular system (10, 40, 78–84), lung (5, 6, 60, 77, 85–89), brain and spinal cord (53, 90–92), intestine (93–96), heart (97), liver (63), skin (98, 99), eye (100, 101), pancreatic islets (102), and many different forms of cancer [reviewed in (103–105)]. The accumulation of versican in these tissues is usually associated with other ECM components that bind versican, such as hyaluronan (106, 107), link protein, TSG-6, IαI, and CD44 (95, 96, 108–111) (Figure 1). Complexes form as a result of this interaction which shape the microenvironment which impacts immunity and inflammation (95, 96, 109–113). Versican is usually found co-localized with hyaluronan, however, inflammatory situations exist where each can be found separately (114, 115). Like versican, hyaluronan is also well-known for having both pro- and anti-inflammatory properties [reviewed in (95, 96, 111, 113, 116, 117)]. It remains to be determined whether the respective pro- or anti-inflammatory activities of versican and hyaluronan are interdependent. Usually, when interventions are used to reduce either versican or hyaluronan in cells and tissues, both components are similarly affected, adding to the challenge of determining the independent contributions of each molecule in the inflammatory process.

Versican interacts with receptors, such as CD44, PSGL-1, TLR2 (118), found on the surface of immune cells, and P- or L-selectins (20, 51, 52, 119–123). This interaction initiates a signaling cascade that influences the phenotypes of immune and inflammatory cells. For the TLRs, versican, like hyaluronan, does not possess the biochemical homology common to ligands for TLR2 and TLR4, such as LPS, so the precise interaction of versican with the TLRs is a bit unclear. No doubt interaction with the TLRs for versican, and maybe hyaluronan as well, involves a complex with molecules that have an affinity for the TLRs (113). In addition, it is interesting to compare the consequences of postulated versican–TLR interactions to other proteoglycans that have peptide motifs that bind TLRs, such as biglycan (18, 124). Whereas biglycan impacts inflammation by activating TLR4 and inflammasomes through activation of Trif-dependent signaling pathways (4, 6, 14, 19, 125), versican appears to mediate TLR2 interaction and activate MyD88-dependent signaling (118, 119). The functional significance of these two separate pathways in governing the inflammatory response is not clear. It will be important to determine if versican has unique immunogenic properties when compared to other ECM molecules.

The negatively charged CS chains in large part control the ability of versican to interact with a multitude of other molecules including chemokines, growth factors and proteases (51, 52, 122, 126–128) impacting their bioavailability and bioactivity (129–131). A number of studies demonstrate that CS chains can also promote the release of proinflammatory cytokines from macrophages and splenocytes, regulate MHCII intracellular trafficking, antigen presentation and T-cell activation [reviewed in (53)].

While a majority of studies show versican as having proinflammatory properties (see below), some studies have indicated that versican can operate in an immunosuppressive manner under certain conditions. For example, Xu et al. (132) found that treatment of mice exposed to LPS with siRNA to versican V1 resulted in increases in TNFα, NFκB, and TLR2 which were accompanied by increases in leukocytes in the lung. Interestingly, we found that LPS stimulated the expression of versican by macrophages in a type I IFN-dependent manner and that deletion of versican from macrophages promoted increased leukocyte invasion in mice exposed to poly I:C (60, 87). These studies are important since they suggest that macrophage-derived versican may possess immunosuppressive characteristics. Additionally, Coulson-Thomas and colleagues showed that the surface of human umbilical cord mesenchymal stem cells contains a glycocalyx enriched in hyaluronan, versican, TSG-6, and IαI, protecting the stem cells from immunodestruction (133) and affecting T-cell and macrophage phenotype. Interestingly, in tumors, human cancer stem cells synthesize and secrete prominent pericellular coat matrices enriched in hyaluronan and versican (134). Such a “cell coat” may account, in part, for the resistance to chemotherapy that these cells exhibit, as well as protection from immune surveillance as has been described for hyaluronan (135, 136) (see below).

## Versican: Interplay With Leukocytes

Leucocytes interact with the ECM as they invade tissue as part of the inflammatory response (5, 7–9, 17, 21) (see Figure 1). To explore this interaction, Carol de la Motte's laboratory at the Cleveland Clinic pioneered the use of co-cultures of leukocytes with stromal cells to explore the mechanisms and consequences responsible for the interaction of leukocytes with the ECM. Using agonists that promote ER stress, these models demonstrated that stromal cells produce an ECM organized into cable like structures that bound leukocytes [see reviews (96, 116, 137)]. These cable-like structures contain hyaluronan, versican, TSG-6, and IαI that bind different subsets of leukocytes (93, 94, 112, 114–116, 137–147). Such structures have not only been found *in vitro*, but also in diseased tissues, such as in atherosclerosis (10, 84), inflammatory bowel disease (IBD) (94), and in brain and spinal cord injury (53, 91). Further studies are needed to determine the contribution of each of the components in the complex on leukocyte phenotype. For example, we found that blocking versican accumulation in the ECM generated by cultured human lung fibroblasts using a neutralizing antibody significantly reduced monocyte adhesion *in vitro* (115). In addition, we showed that manipulating the expression of versican by overexpressing the V3 isoform in experimentally-induced atherosclerotic lesions in rabbits significantly reduced macrophage infiltration, inhibiting the development of lipid-filled atherosclerotic lesions (84). Furthermore, our *in vitro* studies using rat arterial smooth muscle cells demonstrated that the V3 expression effect is anti-inflammatory and decreased the expression of the CS-containing versican isoforms, V0 and V1, and the formation of elastic fibers which are a poor substrate for macrophages (42, 43). Such results further support a critical role for versican in myeloid cell accumulation in atherosclerosis and perhaps in other diseases as well (39).

Lymphoid cells also interact with the ECM. An effective immune response requires that T cells are able to adhere to and migrate through the ECM (7, 145). For example, activated human CD4+ T cells bind to the ECM generated by human synoviocytes and human lung fibroblasts treated with poly I:C, but not to the ECM generated by the stromal cells in the absence of poly I:C treatment (145). This binding blocked the ability of T cells to spread and migrate and was reversed by pretreatment of the ECM with chondroitin ABC lyase. Additionally, versican blocked hyaluronan binding to T cells and inhibited IL-10 synthesis reducing the immunosuppressive capacity of these cells (145). Versican also inhibited human T-cell invasion of collagen gels consistent with influencing T-cell migration and immunosuppression. These activities, of course, could be critical to influencing the ability of T cells to invade and destroy tumor cells (see below). In addition, versican was identified as one of the most upregulated genes in lymphocytes isolated from patients with Sezary syndrome which is a leukemic variant of cutaneous T cell lymphoma. In this setting, versican promoted the invasive and homing capacity of the lymphocytes from these patients (148). Interestingly, the cleavage of versican by ADAMTS enzymes is critical for T-cell trafficking in mouse models of influenza virus infection (149).

Whether versican is intact or degraded may affect its impact on disease cell phenotype. For example, we showed that the G1 fragment of versican promoted extensive ECM cable formation that interconnected adjacent cells and inhibited proliferation, while the intact form had no activity (150). Using the CRISPR/Cas9 system, Hideto Watanabe's group recently developed mice that synthesized versican lacking the ADAMTS versicanase cleavage site (99). They found that accumulation of intact versican in these mice facilitated TGFβ activity coupled to fibroblast proliferation and myofibroblast differentiation and accelerated wound healing. Interestingly, wounds from the versican cleavage-resistant mice contained elevated levels of M1 macrophages and T cells, suggesting in this context that intact versican enhances inflammatory cell infiltration. These studies highlight the importance of ECM molecules such as versican in regulating the ability of both myeloid cells and T cells to invade tissue which is critical to the immune destruction of tumors in many forms of cancer (see below).

## Versican: Expression by Myeloid Cells and the Impact on Inflammation

Versican is also expressed by myeloid cells and upregulated as part of the inflammatory response (see Figure 1). For example, versican expression is elevated by myeloid cells in autoimmunity (127, 151, 152), coronary stenosis (153), myocardial infarction (154), and in response to proinflammatory stimulants such as hypoxia (155, 156) and LPS (60, 87, 157). Versican is differentially expressed in M1 macrophages, as opposed to M2 macrophages, as they differentiate from monocytes (87, 156, 158). On the other hand, a recent study shows that versican enhances mesothelioma growth by promoting M2 polarization and inhibiting phagocytosis (159). More work is needed to sort out whether specific isoforms of versican play a role in determining and/or regulating macrophage phenotype. In patients with systemic sclerosis, CD14-positive monocytes (127) show elevated expression of versican that is accompanied by elevated expression of CCL2 [also known as Monocyte Chemoattractant Protein 1 (MCP-1)]. Interestingly, versican protects CCL2 from degradation which in turn promotes monocyte migration (127). Earlier studies showed that in a model of neuronal inflammation hyperalgesia, CCL2 binds to versican and impacts inflammation (160). A similar relationship has been seen among versican, macrophages, and CCL2 and the promotion of inflammation in mouse models of cancer (161, 162) (see below). Versican also interacts with CCL5 which is important in recruiting CD8 T cells in the inflammatory response (51). Versican has also been identified as a gene common for classical monocytes (CD14^++^ CD16^−^) and classical CD11c dendritic cells (163).

LPS and poly I:C, two TLR agonists, stimulate versican expression in both murine bone marrow-derived macrophages and alveolar macrophages *in vitro* (87) and in murine alveolar macrophages as well as in stromal cells *in vivo* (60, 88). To determine the role of versican derived from macrophages in the innate immune response *in vivo*, we developed two models of conditional versican deficiency by floxing exon 4 of the versican gene. LysM/*Vcan*^−/−^ mice have constitutive myeloid cell-specific versican deficiency (60), and R26R^ert2^Cre^+^/*Vcan*^−/−^ mice, when treated with tamoxifen, are globally deficient in versican (88). *In vitro* studies with macrophages from LysM/*Vcan*^−/−^ mice indicate that versican is important for production of type I IFNs and IL-10 by macrophages in response to poly I:C. This is supported by *in vivo* studies with LysM/*Vcan*^−/−^ mice which indicate that myeloid-derived versican restrains recruitment of inflammatory cells into lungs and promotes production of the key anti-inflammatory cytokines, IFN-β and IL-10, in the pulmonary response to poly I:C. In contrast, *in vivo* studies with R26R^ert2^Cre^+^/*Vcan*^−/−^ mice suggest that stromal-derived versican promotes pulmonary inflammatory cell recruitment in response to poly I:C. When considered in tandem, these results identify versican derived from macrophages as an immunomodulatory molecule with anti-inflammatory properties whereas versican derived from stromal cells has proinflammatory properties. Some of the factors that determine the pro- or anti-inflammatory properties of versican include cellular source, surface receptors, signaling pathways affected, nature of the binding partners that associate with versican and/or whether versican is intact or degraded. It should also be noted that reducing versican by genetic manipulation or other means frequently reduces the accumulation of hyaluronan (88) raising some question as to whether the opposing inflammatory properties of versican may be governed by hyaluronan?

## Versican and Inflammation in Cancer: Pro- and/or Anti-Inflammatory?

Versican is a central player in cancer development in that it impacts tumor-promoting inflammation, immune surveillance evasion, and immunomodulation (164). Versican expression increases as part of the inflammatory response in a number of cancers [reviewed in (22, 103, 105, 165)]. In both breast cancer and Lewis lung carcinoma, the presence of versican produced by the tumor cells leads to an accumulation and activation of tumor-associated macrophages (TAMs) via TLR2 and its co-receptors TLR6 and CD14 (118, 119, 159, 165–167). Tumor cell-derived versican in turn promotes accumulation and secretion of proinflammatory TNFα and IL-6. In addition, we found significantly elevated expression and accumulation of versican in leiomyosarcoma (LMS), a metastatic uterine cancer, when compared to the more benign leiomyomas and control healthy tissue (103, 168). Importantly, cultured human LMS cells synthesized large quantities of versican, forming extensive pericellular coats around the tumor cells. Blocking versican synthesis with siRNA to versican reduced the thickness of the cell coats and inhibited their proliferation and migration *in vitro* and tumor formation in vivo (103, 168).

Stromal cells are a major source of versican as well. In breast and ovarian cancer, TGFβ is overexpressed and contributes to strong stromal versican upregulation (169, 170). Cervical and endometrial cancers are characterized by increases in versican originating from both tumor and stromal cells (171, 172). On the other hand, lack of versican expression in a mouse fibrosarcoma model resulted in a decrease in the number and density of cancer-associated fibroblasts (CAFs) in stroma (173). Such changes led to larger tumors and poorer prognosis in pancreatic cancer (174). In Lewis lung carcinoma, stromal cell-derived versican and its fragment, versikine are associated with increased angiogenesis as part of the inflammatory response contributing to this tumor (175). Accumulation of versican in tumors is positively correlated with the number of microvessels within tumor stroma (176, 177). We found, for example, that human stromal stem cells that produce elevated levels of versican formed an extensive vascular network enriched in hyaluronan and versican when cultured with vascular endothelial cells (178). Furthermore, when patches containing these proangiogenic cells were transplanted onto athymic rat hearts they developed 50-fold more vessels than patches containing stromal cells with low versican expression (178). In other studies, we demonstrated that versican was actively processed in the early stages of VEGF-induced pathological angiogenesis generating extensive DPEAAE (versikine) fragments that associated with the endothelial cells (179). These results suggest that versican, in some form, may be critical for the early stages of angiogenesis as part of the events associated with inflammation.

Myeloid cells are also a major source of versican in tumor inflammation. Versican from myeloid cells promotes tumor metastasis in breast cancer (180). Versican expressed by CD11b+ Ly6C^high^ myeloid cells promotes lung metastasis in a TGFβ-dependent manner in mouse models (181). Intriguingly, versican expression is upregulated by macrophages when co-cultured with carcinoma cells (161, 162), suggesting that the source of versican includes both myeloid cells associated with cancer cells (182, 183). These studies raise the possibility of “crosstalk” among different cell types within the tumor that may influence the nature of versican accumulation and bioactivity which would provide key links to the inflammation associated with cancer initiation, promotion, and metastatic progression (see Figure 1).

Infiltrating myeloid cells secrete versican which can also exhibit immunosuppressive activities in some cancers (74, 75, 103, 118, 184, 185). For example, ADAMTS-generated versikine regulates mouse BATF3-dendritic cell (BATF3-DC) differentiation (74). BATF3-DCs control CD8+ abundance in the tumor microenvironment (186). These observations support a role for versican as being tolerogenic. In addition, a recent study (187) using bone marrow biopsies from 35 myeloma patients revealed a significant correlation of versikine accumulation with infiltration of CD8+ T cells supporting a model in which macrophages and regulatory DCs secrete tolerogenic versican which is subsequently degraded, generating versikine and further altering the immunosuppressive nature of the tumor microenvironment. Thus, taken together, these studies establish that both intact versican and a proteolytic degradation product of versican have immunomodulatory properties and suggest that the anti-tumor properties of versikine might antagonize the pro-tumor properties of intact versican (13, 23, 73, 75, 188). The juxtaposition of these findings indicates that the consequences of ECM-derived–DAMP interactions with PRRs can have sharply differing outcomes depending on the contextual specifics of versican structure.

The tumor stromal ECM microenvironment is characteristic of a wound that does not heal (189, 190) and is functionally analogous to an immune privileged site in normal tissue such as in the eye [reviewed in (191)]. Versican and hyaluronan are enriched in immune privileged sites. The expression and accumulation of versican in the microenvironment of some tumors is associated with reduced numbers of CD8-positive T cells indicating that versican may interfere with T-cell invasion as part of an immunosuppressive activity (192). In addition, versican may be a player in regulating the expression of PD-L1 as part of the autoimmune checkpoint involved in tumor escape from the T-cell immune response. Hartley and colleagues showed that versican produced by mouse tumor cells stimulated monocytes to produce TNFα in a TLR2-dependent manner which in turn upregulated the expression of PD-L1 by mouse monocyte/macrophages (193). The involvement of versican in regulating PD-L1 expression in T cells awaits further investigation. However, such results overall suggest that versican may be part of the exclusionary zone in the microenvironment impacting and preventing T cells access to the tumor (191, 194–196). Such an involvement should be considered as a potential target in immunotherapy treatment of cancer. For example, versican accumulation in the stroma could interfere with T cell-mediated tumor destruction by displacing the T cells from the appropriate tumor target (191).

While the CS containing isoforms of versican appear to be pro-inflammatory, V3 which contains no CS chains when overexpressed in arterial smooth muscle cells inhibits the expression of pro inflammatory cytokines such as CXCL1, CCL20, and CCL2 resulting in blockade of epidermal growth factor receptor and NFκB signaling activity (42, 43). V3 expression also reduced the rate of tumor growth of melanoma by inhibiting tumor cell proliferation as well as increasing the rate of apoptosis. Such experiments highlight a potential role for V3 in counteracting the inflammatory response associated with cancer (197–199).

## Conclusions

Studies indicating a causal role of versican regulating events that drive immunity and inflammation are increasing. There is no doubt that versican, either intact or degraded, as an ECM participant, has a role, but whether it acts alone or in combination with other components during inflammatory events is still not fully understood. Published studies indicate that versican has both pro- and anti-inflammatory activities depending upon the context in which versican is presented to cells. Versican's role in inflammation also depends on temporal and spatial considerations and it may play different roles whether it is involved early or late in disease and whether it is intact or degraded. Furthermore, versican's versatility in binding to multiple receptors and other components involved in the inflammatory response identifies it as a “keystone molecule” regulating inflammation. Developing targeted reagents and therapeutic strategies to interfere with versican accumulation should further identify key mechanisms regulating versican's biological activity. Versican is increasingly being seen as a potential therapeutic target in multiple diseases.

Thus, future directions will be to take advantage of the mouse models we have developed in which versican has been deleted conditionally in the whole animal or specifically in myeloid cells and determine the impact of selectively removing versican in mouse models of cardiovascular and lung disease, autoimmune diseases such as type 1 diabetes and multiple sclerosis, and in some cancers, particularly breast cancer. Such studies have not been possible in the past due to the unavailability of the versican KO mouse. We are interested in focusing on the impact of the different versican variants, such as V3 to better define their role in inflammation and immunity since our preliminary data indicates that V3 acts differently than V0 or V1 in influencing events that drive the immune and inflammatory response. Our cell biology studies will focus on how versican influences immune cells and the immune response focusing on its role in antigen presentation, immune synapse formation, and immune activation important, for example, in the destruction of tumors. Indeed versican, as part of the ECM, is “versatile” and a “keystone” molecule in the regulation of immunity and inflammation.

## Author Contributions

TW wrote the manuscript. IK, SE, IH, MC, OP, CA, and CF edited and revised the manuscript. All authors have reviewed and approved the final version of this article.

### Conflict of Interest

The authors declare that the research was conducted in the absence of any commercial or financial relationships that could be construed as a potential conflict of interest.
